# A soft, bioinspired artificial lymphatic system for interactive ascites transfer

**DOI:** 10.1002/btm2.10567

**Published:** 2023-08-03

**Authors:** Peng Wang, Ji Fu, Peng Jin, Jin Zeng, Xiaohui Miao, Heling Wang, Yinji Ma, Xue Feng

**Affiliations:** ^1^ AML, Department of Engineering Mechanics Tsinghua University Beijing China; ^2^ Laboratory of Flexible Electronics Technology Tsinghua University Beijing China; ^3^ Institute of Flexible Electronics Technology of THU Jiaxing Zhejiang China

**Keywords:** artificial lymphatic system, ascites transfer, flexible electronics

## Abstract

Low‐flow removal of refractory ascites is critical to treating cirrhosis and digestive system tumor, and thus, commercial ascites pump emerged lately. The rigid structure of clinically available pumps rises complication rate and lack of flow rate monitoring hinders early warning of abnormalities. Herein, a soft artificial system was proposed inspired by lymph for interactive ascites transfer with great biocompatibility. The implantable system is composed of pump cavity, valves and tubes, which are soft and flexible made by silica gel. Therefore, the system possesses similar modulus to tissues and can naturally fit surrounding tissues. The cavity with magnetic tablet embedded is driven by extracorporeal magnetic field. Subsequently, the system can drain ascites with a top speed of 23 mL min^−1^, much higher than that of natural lymphatic system and state‐of‐art devices. Moreover, integrated flexible sensors enable wireless, real‐time flow rate monitoring, serving as proof of treatment adjustment, detection and locating of malfunction at early stage. The liver function of experimental objects was improved, and no severe complications occurred for 4 weeks, which proved its safety and benefit to treatment. This artificial lymphatic system can serve as a bridge to recovery and pave the way for further clinical research.

## 1 INTRODUCTION

Ascites refers to a pathological accumulation of fluid within the peritoneal cavity and is the most common complication of cirrhosis.^1^, ^2^ The widely accepted hypothesis of ascites pathophysiology is that cirrhosis increases intrahepatic resistance and consequently reduces lymphatic flow. Up to 70% of patients will develop ascites in the first 10 years after the diagnosis of cirrhosis, and approximately 11.4% of them will eventually develop refractory ascites (RA) with an unsatisfactory response to diuretic therapy and sodium restriction.^1^, ^3^ Patients with RA usually suffer from malnutrition, sarcopenia and impairment of health‐related quality of life, with the 1‐year overall survival rate of RA being only approximately 50%.^4^, ^5^

Currently, the available clinical treatments for ascites are large‐volume paracentesis (LVP) and transjugular intrahepatic portosystemic shunt (TIPS). LVP serially drains ascites from the peritoneal cavity to relieve discomfort, and the reported complication rate of the procedure is relatively low. However, the process directly causes an abrupt large volume removal of ascites and induces cardiovascular and hemodynamic disorders. Additionally, ascites reaccumulation between treatments and frequent hospitalization have a negative effect on health‐related quality of life.^6^ TIPS attempts to treat RA pathogenically by releasing the increased portosystemic pressure. Previous studies have proven that TIPS has a lower procedural failure rate and paracentesis frequency than LVP at the cost of more hepatic encephalopathy cases reported. Additionally, successful completion of TIPS greatly depends on the expertise and skills of surgeons, thus restricting its application.

Recently, automated low‐flow pump (alfapump) was proposed as a promising solution to complications of conventional methods.^7^, ^8^ Alfapump is a subcutaneously implanted and rechargeable device that can automatically drain ascites into the urinary bladder and void the fluid with urine. The low transfer rate efficiently eliminates paracentesis‐induced circulatory dysfunction and reduces hospitalizations, thus improving health‐related quality of life. Nevertheless, the rigid and incompact structure of the device accounts for a high rate of adverse events reported thus far. Abdominal pain occasionally occurred, led to pump failure, and even resulted in a second surgery.^9^, ^10^ In addition, the commercial pump lacks flow rate monitoring, hindering early warning of abnormalities, such as gear and duct blockage. Therefore, a new design with perception is demanded to meet the flexibility and biocompatibility in this scenario.

Lately, soft machines have been widely researched for biomedical applications^11^ and, fortunately, flexible electronics technology has provided a solution to integrated perception of surroundings,^12^, ^13^, ^14^, ^15^, ^16^, ^17^, ^18^, ^19^, ^20^, ^21^, ^22^ which has been widely used in healthcare devices,^23^, ^24^, ^25^, ^26^, ^27^ implants^28^, ^29^, ^30^, ^31^, ^32^, ^33^, ^34^ and soft machine.^35^, ^36^ Herein, we propose a soft artificial lymphatic system for interactive ascites transfer as a compensation for inadequate or impaired lymphatic function in patients with RA. The duckbill valves and pump cavities mimic lymphatic valves and follicles, respectively, and restrain potential counterflow. Inspired by the lymph, the system is actuated by the movement of an internal magnetic tablet in an external magnetic field. Furthermore, the real‐time flow rate can be detected by cuff‐type sensors placed along the duct and used as a feedback to adjust treatment period. Additionally, the flow rate can provide proof of malfunction occurrence and position at early stage. The operation of the artificial lymphatic system is illustrated in vitro by fluid transport and in vivo in a pig model, leading to no severe complications during 1‐month experiment and ascites transfer function with palliation of liver function.

## 2 RESULTS

### 2.1 Design of the artificial lymphatic system

**FIGURE 1:**
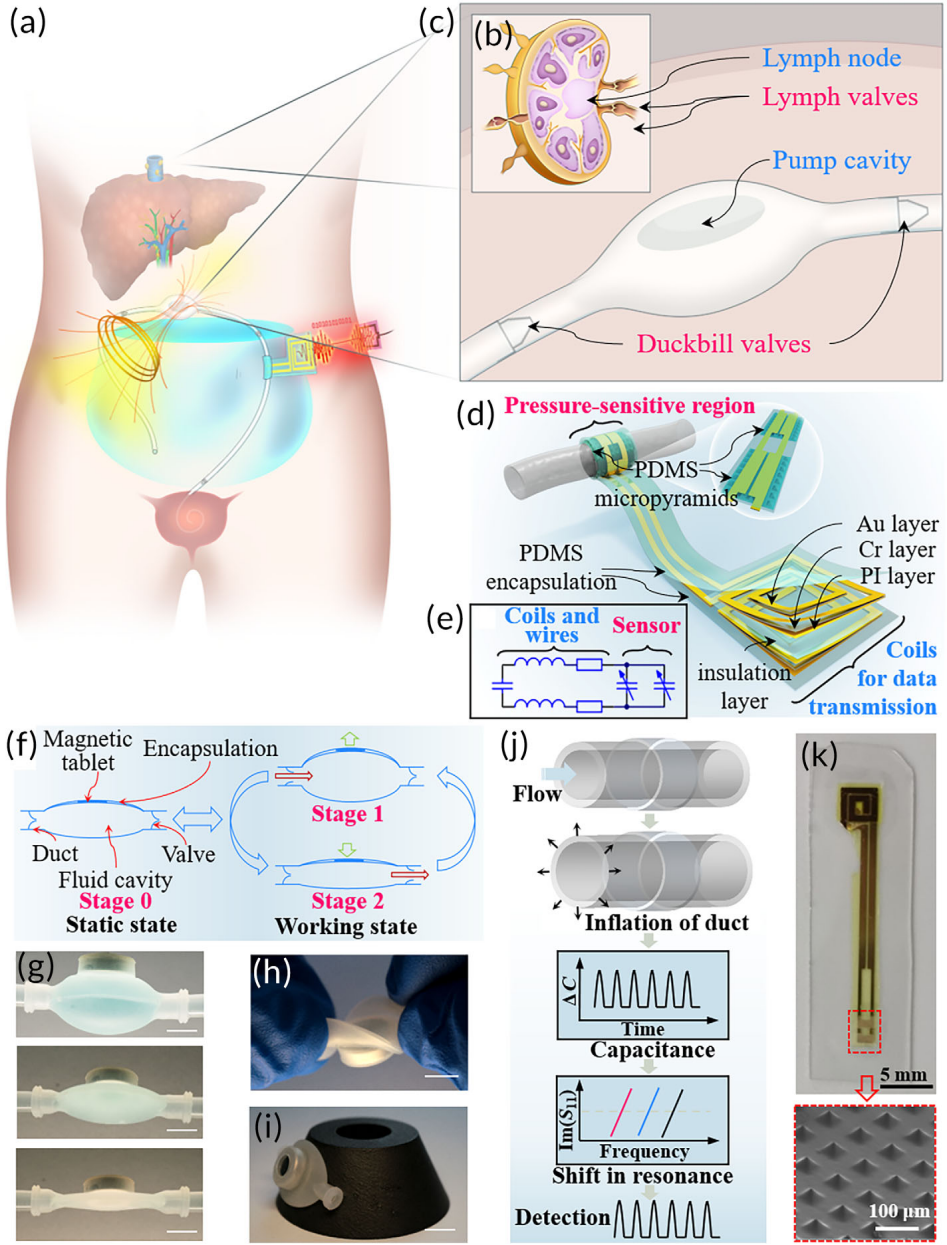
Smart flexible artificial lymphatic system design. (a) The artificial lymphatic system can automatedly drain ascites into the urinary bladder. (b) Structure of the lymphatic system. (c) Structure of the artificial lymphatic system. The lymphatic system consists of a lymph node and valves that can collect and direct the lymphatic fluid, and the artificial lymphatic system consists of a pump cavity and duckbill valves, showing great resemblance in structure. (d) Illustration of the sensor with an exposed view of the bilayer coil structure for wireless communication and the cuff‐type capacitive sensor mounted around the duct. The sensor is encapsulated and insulted by a PDMS layer, and an equivalent inductance is formed between two coils. The inset in d shows that micropyramids are fabricated as a pressure‐sensitive region and that two variable capacitors are separately connected in parallel. (e) Equivalent electrical circuit. The two variable capacitors correspond to two pressure‐sensitive regions in d. The two inductors and the fixed capacitor correspond to the top and bottom coils, and the PDMS insulation layer, respectively. The fixed resistances correspond to the resistances of wires. (f) Actuating principle. The internal electromagnet was actuated by alternating magnetic field and drained ascites in and out the pump cavity. (g–i) Optical images of the pump cavity (g) in operation, (h) in a mixed mode of folding and twisting, and (i) on a curved surface. (Scale bar: 1 cm). (j) Sensing principle. The ascites flow driven by magnetic actuation leads to a change in duct diameter, which can be detected by a pressure‐sensitive region around the duct. The pressure‐sensitive region can convert dilation into capacitance changes and, combined with an inductor coil, form an LCR circuit. The shift in resonant frequency caused by capacitance changes is wirelessly measured through the abdominal wall by an external reader coil. (k) Image of a fabricated device (top) and scanning electron microscope image of the pressure‐sensitive region showing PDMS micropyramid structures (bottom).

The artificial lymphatic system actuated by an external magnetic field can drain ascites from the abdominal cavity for treatment of RA (Figure 1a). Previous studies have proven that bioinspired pump designs can control the average flow rate, but they need to prevent diffusion and backflow of urine. Therefore, inspired by lymphatic valves, duckbill valves were introduced into the system to guide ascites through the ducts to the urinary bladder. Lymphatic circulation is driven by muscular movement, such as vascular pulsation and intestinal peristalsis, so periodic pressure was applied on the pump cavity as the driving force of the artificial lymphatic system.^37^, ^38^ After the pumps were subcutaneously implanted, various failures were reported, such as duct blockage and abdominal pain. Most of the abnormities could be reflected by unexpected fluctuations in the flow rate, and thus, wireless real‐time detection of the flow rate can inform the system about its own operating status. Resembling the lymphatic structure (Figure 1b), the proposed artificial lymphatic system consists of a pump cavity, duckbill valves, ducts for connection and additional wireless flow rate sensors (Figure 1c). All of the compositions are made of soft silica gel, whose modulus is around 20 kPa^39^ and similar to that of human tissues, thus can reduce discomfort and avoid severe pain. An external magnetic field was applied to actuate an internal magnetic tablet and enlarge the controllable range of the flow rate. Flexible sensors were placed at the outlets of duckbill valves and communicated with another coil for data transmission.

The sensor was fabricated by a benchtop process including lamination, lithography, transfer printing and packaging with biocompatible silica gel. Ten nanometer‐thick nickel (Ni) and 150 nm‐thick gold (Au) were laminated on a silicon wafer with polymethyl methacrylate (PMMA) as the sacrificial layer and polyimide (PI) spun over. After lithography, the patterned laminate was transferred onto a polydimethylsiloxane (PDMS) substrate for better flexibility (Figure S1). The patterned laminate was electrically interconnected with a 100 μm‐thick microstructured PDMS dielectric layer as a pressure‐sensitive region (Figure 1d). The PDMS dielectric layer with a pyramid microstructure was shaped over silicon micromolds, and this design has been proven to improve the sensitivity and response time of the sensor. The inductor–capacitor–resistor (LCR) circuit (Figure 1e) was assembled by a lamination process instead of establishing additional electrical interconnections between two coils. As previously reported,^40^ the asymmetrical assemblage was demonstrated to enlarge the resonance shift when the same capacitance change was applied. When the two coils were laminated, a layer of PDMS was inserted in the middle as an insulation layer to avoid short circuits. Since the sensor is encapsulated by a stretchable polymer and the total thickness is only several hundred microns, it possesses intrinsic flexibility compared to conventional devices. The stiffer laminate structure was wrapped around the pressure‐sensitive region when the sensor was assembled onto the duct. The relatively high stiffness of the laminate structure compared to the encapsulation layer makes the sensor more sensitive to flow rate changes rather than surrounding muscle movement.^40^

The actuating principle is demonstrated in Figure 1f. When no actuation is applied, the pump cavity remains static in Stage 0. After the alternating magnetic field is applied, the cavity periodically varies between two stages, and the changes in fluid pressure in the cavity can also be split into two stages. During Stage 1, the magnetic tablet moves toward the external electromagnet, and the volume is enlarged. A negative pressure is created inside and makes the inlet valve open. Once the cavity is filled with the fluid, the differential pressure fails to maintain the state of the inlet valve, and the pump cavity stops taking in fluid. During Stage 2, the magnetic tablet moves in the reverse direction, and increased pressure opens the outlet valve. The fluid is squeezed out until the pressure is lower than that at the outlet. After the alternating magnetic field is removed, the pump cavity changes back to the static state, Stage 0. The optical images of the cavity in operation are shown in Figure 1g.

The pump cavity was fabricated by simple injection molding. Two concentric semiellipsoid molds, which were made of 400C steel to avoid potential allergens, were used to define the geometry of the elements. Two elements were connected by silica gel to assemble a pump cavity after injection molding using biocompatible silica gel. The flexible pump cavity was attached to a magnetic tablet with a biocompatible binder and then encapsulated by a thin layer of silica gel. The cavity was connected to duckbill valves by medical‐grade ducts, which were also made of biocompatible silica gel by injection molding to form the main part of the artificial system. The pump cavity possesses inherent flexibility in a mixed mode of folding and twisting (Figure 1h), and on a curved surface (Figure 1i).

The sensing principle is illustrated in Figure 1j, and the structure is shown in Figure 1k. When the flow went through them, the ducts were inflated under the influence of differential pressure and pump actuation. The fringe‐field capacitor of the sensors could reflect the inflation through the change in capacitance whether the sensors contacted the ducts. Combined with the coils, the whole sensor is equivalent to an LCR circuit, whose shift in resonant frequency depends on the synergetic effect of the changes in inductance and capacitance. The resonant frequency is wirelessly detected through the abdominal wall via mutual inductance coupling between the sensor and the external reader coil. Since the artificial lymphatic system is driven by an external magnetic field and the detection is based on mutual inductance, the system is battery‐free and thus vastly superior in reliability to conventional implanted pumps. Details of the processing procedures are shown in the Methods and Figure S2.

### 2.2 Pump cavity design and optimization

**FIGURE 2:**
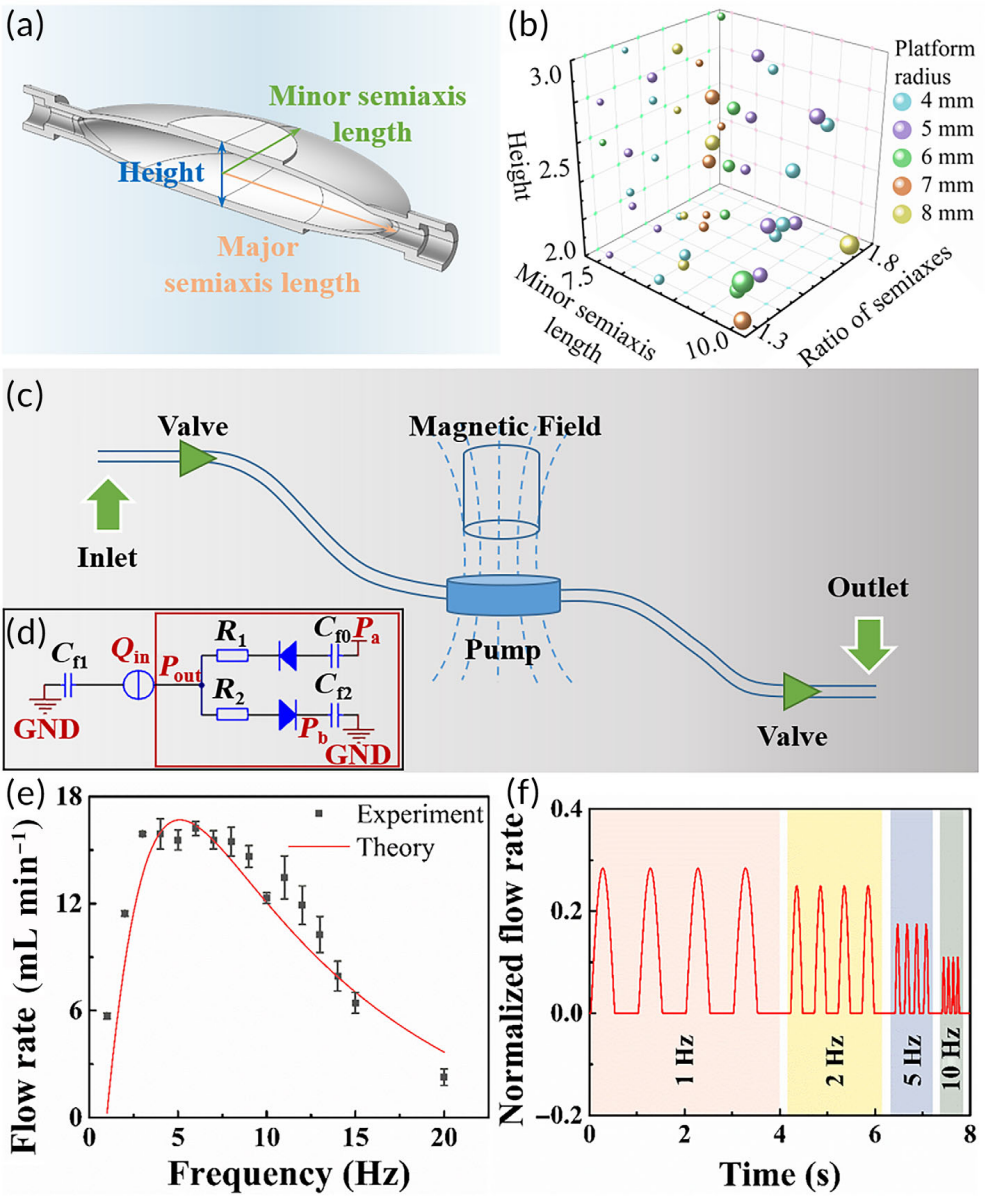
Pump cavity design and optimization. (a) Geometry of the pump cavity. The pump cavity can be seen as an ellipsoid with a platform on top for attachment to the magnetic tablet. The height, major semiaxis length and minor semiaxis length are three main parameters for optimization. (b) Flow rates for the pump cavity with different parameters calculated by the FEM. (c) Illustration of the artificial lymphatic system. The pump cavity can be actuated by an external magnetic field and drain the fluid from the inlet to the outlet under the direction of valves. (d) Equivalent electrical circuit. The actuation is equivalent to a constant current source, and the valves are equivalent to diodes. The capacitors and resistances represent the compliance effect and frictional losses, respectively. (e) Experimental and theoretical efficient flow rate corresponding to the actuation frequency. For both experimental and theoretical results, the efficient flow rate first increases and then decreases with the actuation frequency, and the optimum frequency is 5 Hz. Data are shown as the mean ± SD (*N* = 3). (f) Real‐time normalized flow rate under different actuation frequencies. The fluctuation of the normalized flow rate reduces with the frequency, showing characteristics similar to a low‐pass filter.

The pump cavity can be simplified as an ellipsoid with a platform on top for attachment to the magnetic tablet (Figure 2a). To determine the geometry that can generate the highest flow rate, a series of parameters were analyzed by the finite element method (FEM) using COMSOL 5.6 (COMSOL, Inc.). The geometry was optimized according to orthogonal test designed by SPSS (QingSi Technology, Ltd., China). The main parameters that determine the geometry are planform radius, the ratio of major to minor semiaxis, minor semiaxis length and height. The values of these parameters were set according to previous FEM results. The combinations of these parameters were formed by SPSS (Table 1) and used as input for FEM calculation. The magnetic force was simplified as a uniformly distributed load over the planform and quasi‐state loading. Flow rate was calculated for each combination and the results are summarized in Figure 2b, and the three‐dimensional fluid field is shown in Figure S3. Different colors refer to different planform radii and the size of spheres represents flow rate. Additionally, the projection of the spheres onto three reference surface is also shown in Figure 2b. The projection is uniformly distributed in each reference surface, which is the characteristics of orthogonal test. Afterward, the statistical significance of these results was analyzed regarding different parameters (Table 2). The ratio of major to minor semiaxis, planform radius and height account for statistical significance, while minor semiaxis length is not statistically significant (*p* < 0.05). To choose the optimum geometry of the pump cavity, multiple comparison was performed. According to the results, the radius of the platform was set as 6 mm. An enlarged platform brings larger loading surface and actuation, but restricts the movement displacement and efficient flow rate. Therefore, an optimum exists for the size of the platform. The height, major and minor semiaxis length of the optimum geometry are set as 2.2, 18, and 10 mm, respectively.

**TABLE 1:** Parameter combination design.

Number	Platform radius (mm)	Major semiaxis length (mm)	Ratio of major to minor semiaxis	Height (mm)
1	8	8	1.5	3
2	4	9.5	1.8	2.6
3	6	10	1.3	2.2
4	4	8.5	1.8	2.8
5	5	7.5	1.3	2
6	4	7.5	1.6	2
7	6	7.5	1.3	2.6
8	5	7.5	1.5	2
9	4	8	1.3	2.4
10	5	9.5	1.7	2.4
11	8	9.5	1.3	2.8
12	7	10	1.3	2
13	6	8	1.7	2
14	5	8	1.8	2.2
15	5	10	1.4	2.4
16	4	9.5	1.6	2.2
17	5	10	1.6	2.8
18	5	8.5	1.3	3
19	8	10	1.8	2
20	5	9	1.6	3
21	7	7.5	1.7	2.8
22	5	7.5	1.4	2.2
23	8	9	1.3	2.4
24	4	8.5	1.3	2
25	8	7.5	1.6	2.6
26	6	9	1.5	2.8
27	5	8	1.3	2.6
28	7	9	1.4	2.6
29	4	9	1.3	2.2
30	5	8.5	1.7	2.6
31	7	9.5	1.3	3
32	5	9.5	1.5	2
33	4	7.5	1.3	2
34	4	10	1.5	2.6
35	8	8.5	1.4	2
36	7	8.5	1.5	2.2
37	4	9	1.7	2
38	4	8	1.4	2.8
39	4	7.5	1.5	2.4
40	6	9.5	1.4	2
41	6	7.5	1.8	3
42	7	7.5	1.8	2.4
43	4	10	1.7	3
44	5	9	1.8	2
45	4	7.5	1.4	3
46	5	7.5	1.3	2.8
47	6	8.5	1.6	2.4
48	7	8	1.6	2
49	8	7.5	1.7	2.2

**TABLE 2:** Statistical significance of these results.

Parameters	Degree of freedom	Statistical significance
Platform radius	4	0.128
Major semiaxis length	5	0.000
Ratio of major to minor semiaxis	5	0.373
Height	5	0.050

Hence, to further evaluate the transfer efficiency of the system after implantation, the simplified system (Figure 2c) is described by an equivalent circuit model (Figure 2d). The locomotion mode of the magnetic tablet in the alternating magnetic field is described by the equivalent stiffness (*k*), coupling coefficient (*K*), and alternating voltage (*U*) applied onto the electromagnet. Pump actuation is induced by displacement of the magnetic tablet and can thus be seen as a current source in the fluidic circuit model. The fluidic circuit model for the proposed system is established by fluidic resistances, membrane compliances, and flow rates. Fluidic resistances are used to represent frictional losses in the ducts. Under the dynamic flow rate, the volume change is illustrated by the compliance effect. The valves are embodied by diodes with a negligibly low forward voltage drop. Because the urinary bladder possesses compliance, the vesical pressure *P*_out_ continuously increases until voiding. Without the valves, back flow may be generated from continuous original urine accumulation. Additionally, without magnetic actuation, the current source of the system is seen as an open circuit, and the system is simplified as a series circuit (inside the red rectangle in Figure 2d). When the vesical pressure *P*_out_ reaches abdominal pressure *P*_a_, the flow rate drops to zero owing to valve occlusion. Therefore, external magnetic actuation enlarges the controllable flow rate range of the system. According to the equivalent circuit model, the flow rate of the system is analytically derived as follows:

$$Q_{i}=\left\{\begin{array}{c} -\frac{4}{\pi }\frac{\mathrm{KUS}}{m}R_{1}\sum_{i=1}^{\infty }\frac{\omega_{i}}{\left.\omega_{n}(\omega_{n}^{2}-\omega_{i}^{2}\right)}\frac{1}{\sqrt{1+\omega_{i}^{2}\tau^{2}}}\sin\left(\omega_{i}t-\alpha \right)\;\frac{\alpha }{\omega_{i}}-\frac{T}{2}<t<\frac{\alpha }{\omega_{i}}\\ \;0\;\frac{\alpha }{\omega_{i}}<t<\frac{\alpha }{\omega_{i}}+\frac{T}{2} \end{array}.\right.$$

In this equation, τ indicates the time constant which is expressed as $\tau =\frac{C_{f0}+C_{f2}}{C_{f0}C_{f2}}\left(R_{1}+R_{2}\right)$ and $\alpha =\tan^{-1}\left(\omega_{i}\tau \right)$. Details of the derivation of the flow rate are presented in Note S1 for the system under periodic magnetic actuation.

Obviously, the ascites transfer process includes two strokes, defined as the intake stroke and exhaust stroke, within one cycle. During the intake stroke, the ascites is drained into the pump cavity and the outlet flow rate is zero due to the unidirectionality. During the exhaust stroke, the ascites stored in the pump cavity during the last stroke is squeezed into the urinary bladder.

The efficient flow rate over a cycle showed a strong relation to the actuation frequency both theoretically and experimentally (Figure 2e). For high frequency, the magnetic tablet vibrates slightly around the equilibrium position, which cannot give full play to both strokes. For low frequency, although the motion of the magnetic tablet is free of restriction, it could be completed within a much shorter period. Thus, the optimum frequency for the implanted system is set as 5 Hz.

The compliance effect of the system reduced the fluctuation of the flow and made the system act as a typical low‐pass filter. Thus, the implanted system showed a flow stabilization effect. To evaluate this effect, the real‐time flow rate was calculated with respect to various actuation frequencies, as shown in Figure 2f. Apparently, the fluctuation of the flow rate decreased with the frequency. Regarding the results, the flow rate remained nearly consistent for frequencies higher than 10 Hz. Consequently, this implies that the implanted system behaved as a low‐pass filter.

### 2.3 Sensor characterization and layout

**FIGURE 3:**
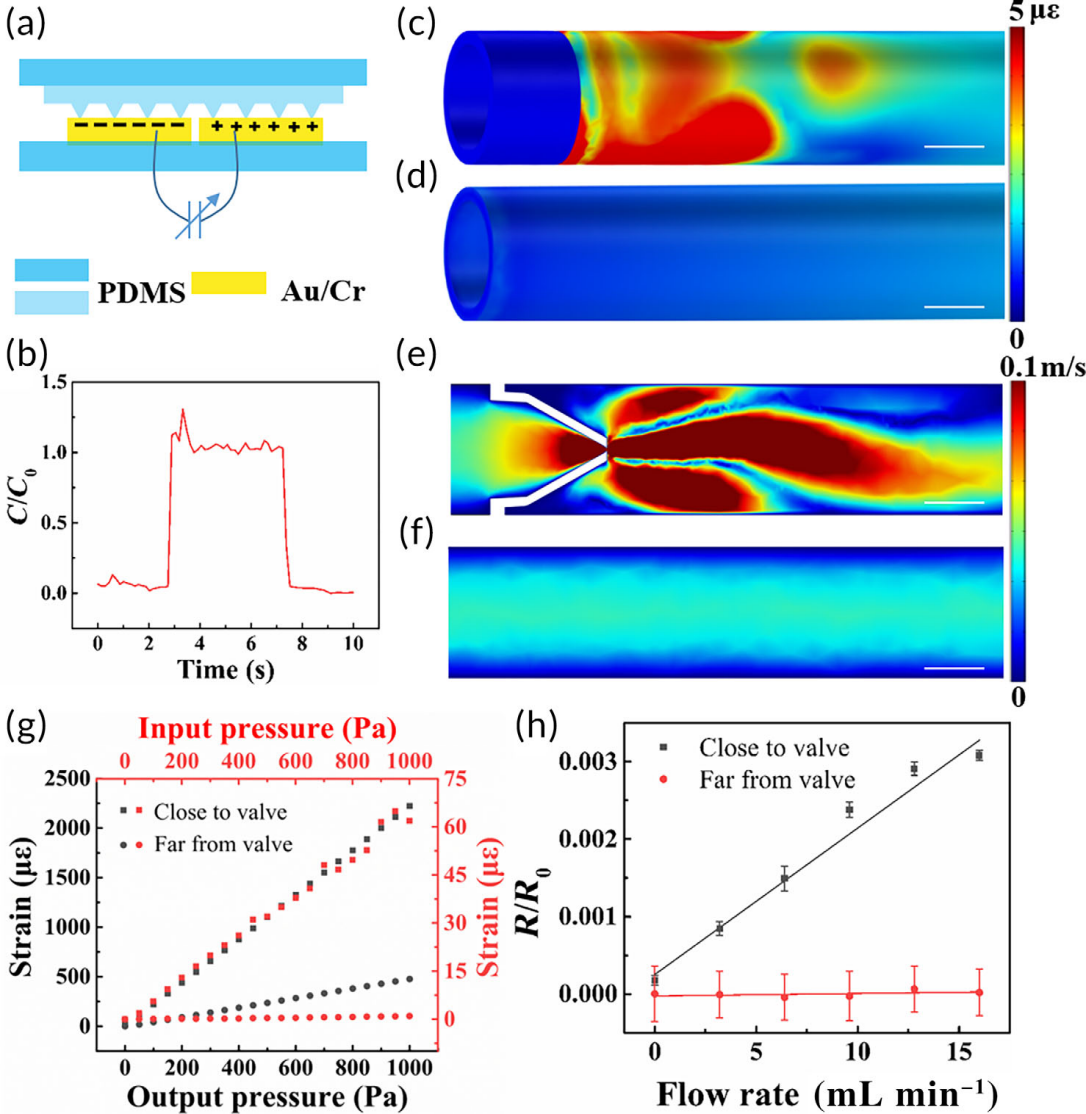
Sensor characterization and layout. (a) Sensor design. The cross‐sectional view is shown in (a), and the induced charge on the two electrodes varies with the pressure, forming a variable capacitor. (b) Sensor response time when pressure was applied and released. (c and d) The main strain close to the valve is more than twice as large as that far from the valve. (c) FEM results of the main strain close to the valve in the duct obtained by COMSOL. (d) FEM results of the main strain far from the valve in the duct obtained by COMSOL. (e and f) The laminar flow input changes in the range close to the valve and back to laminar flow when it is fully developed. (e) FEM results of the flow velocity close to valve in the duct obtained by COMSOL. (f) FEM results of the flow velocity far from valve in the duct obtained by COMSOL. (g) FEM results of the main strain close to and far from the valve in the duct obtained by COMSOL under different input and output pressure. The main strain close to the valve is much higher than that far from the valve. (h) Strain close to and far from the valve measured by flexible film strain grids. The strain close to the valve is confirmed to be larger than that far from the valve in the duct. Data are shown as the mean ± SD (*N* = 3). Scale bar in (c–f), 1 mm.

The cross‐sectional view of the sensor is shown in Figure 3a. When a pressure is applied or an object moves closer, the capacitance between two electrodes changes. For the LCR circuit, the shift in resonant frequency Δ*f* caused by the change in capacitance Δ*C* can be calculated with the following equation:

$$∆f=-\frac{∆C}{4\pi \sqrt{LC^{3}}},$$

*L* and *C* in the equation refer to the inductance and capacitance of the circuit, respectively. Thus, the shift in resonant frequency shows great linearity with the change in capacitance. The inductance of the sensor was 6.93 pH. The response time of the capacitor is shown in Figure 3b and was reduced to the order of milliseconds based on experimental results. This fast response time meets the demand of real‐time monitoring of the flow rate, which can enable warning of abnormalities in an early stage.

Since the sensor detects the flow rate through dilation of the ducts, which differs with location, the layout of the sensor strongly affects the sensitivity. Thus, the main strain and flow velocity in the duct close to and far from the valve were analyzed by the FEM. Obviously, the main strain close to the valve (Figure 3c) is more than twice as large as that far from the valve (Figure 3d). Additionally, the laminar flow input changes in the range close to the valve (Figure 3e) and back to laminar flow when it is fully developed (Figure 3f). These results were confirmed under different input and output pressure, as shown in Figure 3g. The strain close to valve is much larger than that far from valve under any pressure, and placing sensors close to valve can efficiently improve their sensitivity. The strain increases with the input or output pressure, which can distinguish upstream blockages from downstream ones.

To confirm the FEM results, flexible film strain grids were wrapped around the duct close to and far from the valve to directly evaluate the strain (Figure 3h). For the film strain grid placed close to the valve, the relative change in resistance linearly increased with the flow rate, while for the grid far from the valve, the relative change in resistance could barely be observed. Hence, the sensitivity of the sensor is greatly improved by the optimum layout close to the valve.

### 2.4 In vitro characterization of the system

**FIGURE 4:**
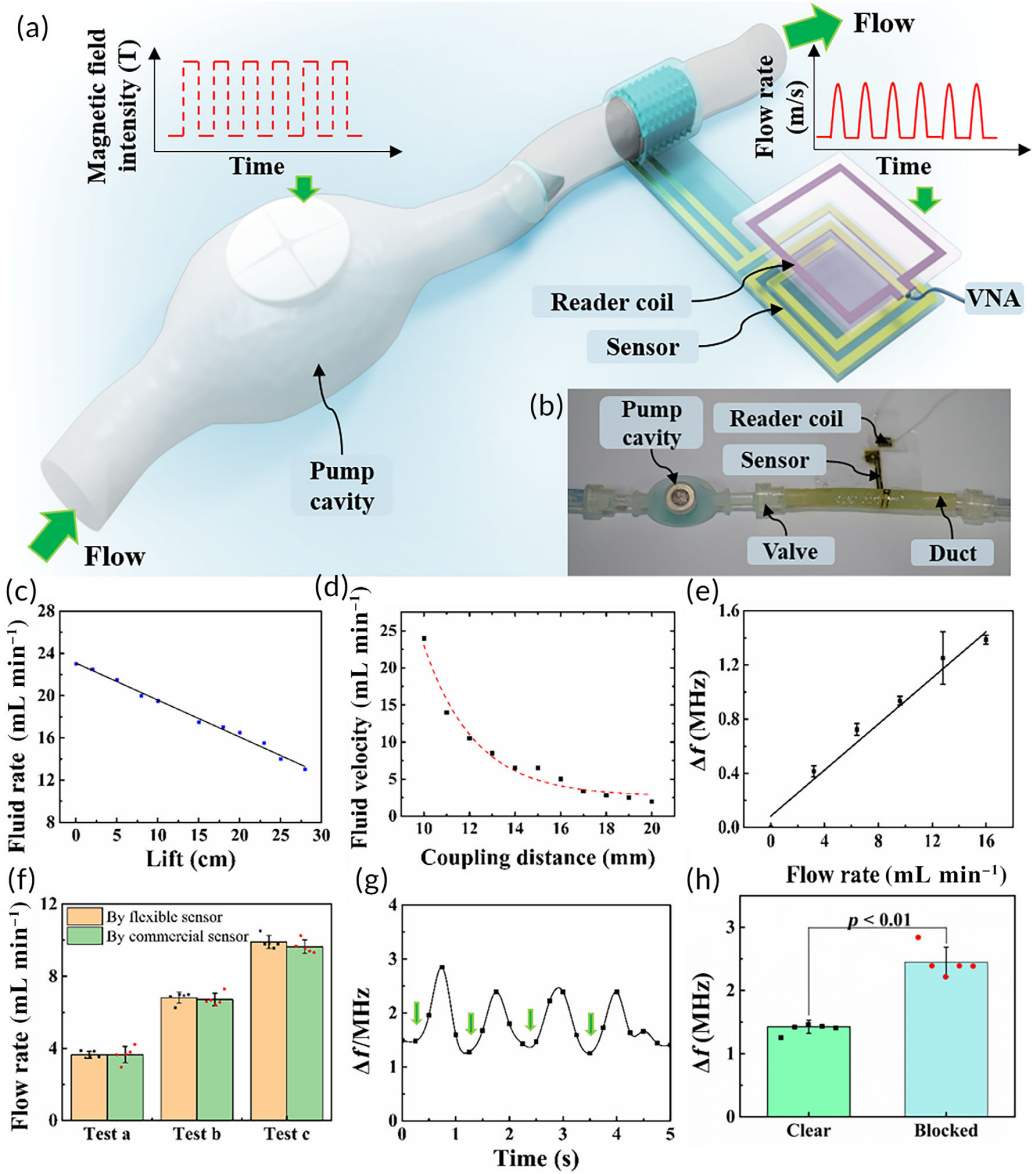
In vitro characterization of the system. (a) Schematic drawing of the experimental setup. An alternating magnetic field was used as actuation to the flow. The reader coil communicates with the internal coil by induction and connects to a VNA for real‐time wireless recording of the shift in resonant frequency Δ*f*. (b) Response characteristics of the pump cavity for different lifts. The flow rate shows a linear negative correlation with the lift. (c) Response characteristics of the pump cavity for different coupling distances. The flow rate is negatively correlated with the coupling distance due to the reduced magnetic field intensity. (d) Sensor calibration. The sensor shows great linearity, and mapping of the shift in resonant frequency and flow rate can be obtained. (e) Comparison of flow rates measured by the flexible sensor and a commercial sensor. Dots represents all data points. Error bars represent ±SD (*N* = 5). (f) Real‐time response to continuous blockage. Green arrows point at the moment when pressure was applied, and an obvious increase can be observed once pressure was applied. (g) Comparison of shifts in the resonant frequency for clear and blocked ducts. Data are shown as the mean ± SD (*N* = 5).

The artificial lymphatic system was first characterized in vitro to better understand its function. The pump cavity was fixed onto the platform and actuated by an electromagnet above it (Figure 4a), and the picture of the in vitro experimental system is shown in Figure 4b. Deionized water was chosen as the ascites simulant, and the inlet and outlet of the artificial system were both placed in open containers. Because the location of the artificial system and the actuation are two easy‐to‐control parameters, the lift and the coupling distance (illustrated in Figure S4) were qualitatively analyzed, as shown in Videos S1 and S2. The flow rate linearly decreased with the lift (Figure 4c) because the lift caused a reverse pressure difference under gravity and reduced efficient actuation. Similarly, the flow rate showed an obvious negative correlation with the coupling distance (Figure 4d) because of the negative relationship between actuation and the coupling distance.

The reader coil connected to a vector network analyzer (VNA) can wirelessly provide the real‐time resonant frequency (*f*_0_) of the sensor by measuring the scattering parameter *S*_11_. To calibrate the sensor, a standard gear pump with a maximum flow rate of 16 mL min^−1^ was selected to replace the pump cavity in the artificial system. The shift in resonant frequency of the sensor with the flow rate was analyzed, as illustrated in Figure 4e. A mathematical fitting was conducted, and the sensor showed great linearity. Additionally, the mapping of the frequency shift with the flow rate was directly obtained. The accuracy of the sensor was confirmed in contrast with a commercial flow rate detector (Figure 4f). No obvious temperature variation was observed for the sensor during the test, as shown in Figure S5.

For artificial systems, the most common abnormality may be blockage of the ducts after implantation. Thus, blockage was mimicked by pinching the duct to stop the flow. The real‐time shift in resonant frequency is shown in Figure 4g. The green arrows point to the moment when pressure was applied. Once the pressure was exerted downstream from the sensor, a jump in the shift in the resonant frequency occurred, which was an obvious and straightforward warning of abnormality (Figure 4h). For blockages formed upstream, the shift in resonant frequency gradually decreases to an observable degree in accordance with the reduced flow rate. Common commercial sensors usually detect the flow rate via the fluid volume passing through their cross section. Thus, wherever the blockage is formed, the flow rate will be measured as zero. Different from commercial sensors, the flexible sensor can distinguish blockage formation positions. Furthermore, the suspicious blockage position can be narrowed to a specific duct by placing a set of sensors, greatly shortening the time for positioning.

### 2.5 In vivo ascites transfer test

**FIGURE 5:**
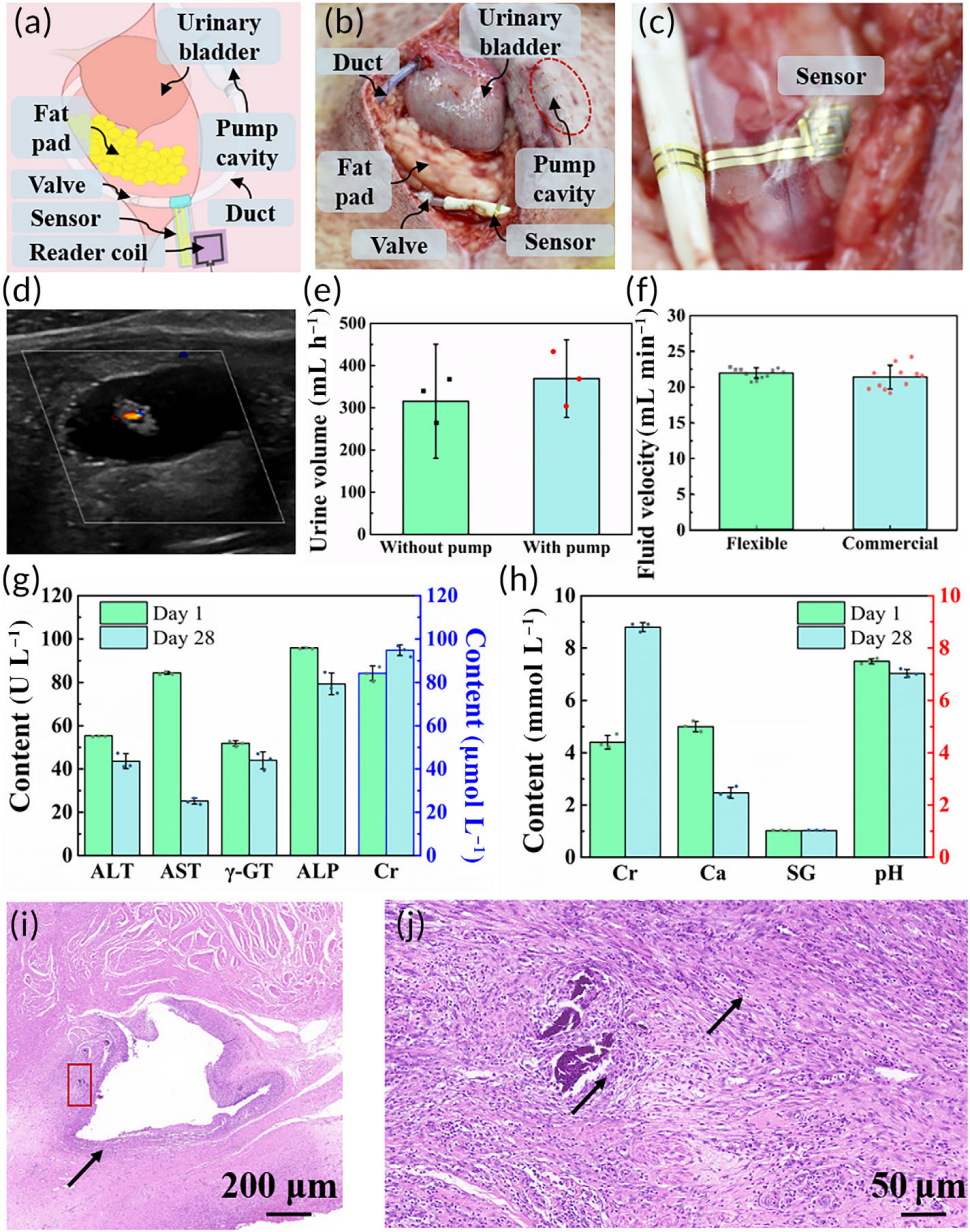
In vivo ascites transfer test. (a–c) Illustration of the experimental setup in a pig. (a) Schematic drawing of the implantation site. (b) Image of the implantation site with the duct inserted into the urinary bladder and fixed with sutures. The pump cavity was placed and fixed inside a pouch beneath the skin, and the wireless sensor was mounted around the duct. (c) The sensor was conformal to the surrounding tissue without any wrinkles. (d) Doppler ultrasound image of the urinary bladder. The outlet of the duct can be observed as a bright gray ring with obvious flow inside. (e) Comparison of urine volumes per hour with/without the pump working. When the pump cavity was actuated by an external magnetic field, the urine volume increased. Data are shown as the mean ± SD (*N* = 3). (f) Comparison of flow rates measured by flexible and commercial sensors in vivo. The results show great consistency for both sensors. Dots represents all data points. (g) Blood biochemical indexes. Main indexes of liver function: ALT, alanine aminotransferase; AST, aspartate aminotransferase; γ‐GT, gamma‐glutamyltransferase; ALP, alkaline phosphatase; Main index of renal function: Cr, creatinine. (h) Urine biochemical indexes. Ca, urine calcium; SG, specific gravity; BLD, occult blood. (i and j) Histopathological section of the urinary bladder. (j) presents an enlargement of the red rectangle in (i). The duct was covered by a great deal of connective tissue with inflammatory cell infiltration (black arrows).

The smart artificial lymphatic system was implanted into a pig to verify its operation, as illustrated in Figure 5. The in vivo experiments were performed under anesthesia by laparotomy and after suture to confirm the effect of the implanted system. The experimental setup is shown in Figure S6 and Video S3. The implanted pumping actuation by the external magnetic field inside the pig's abdomen before suturing it is shown in Video S4.

In vivo ascites transfer was performed in a pig by laparotomy, as shown in Figure 5a–c. The outlet of the duct was inserted into the urinary bladder and the ducts were sutured onto the abdomen at intervals. The pump cavity was placed inside a pouch beneath the skin. The relatively small implantation depth ensured efficient coupling and sufficient actuation. The implanted sensor spread conformally in the tissue, as shown in Figure 5c, which ensured communication with external reader coils. Indigo carmine, a biocompatible coloring agent, was added into ascites simulant and its appearance in urine (Figure S7) could indicate of smooth operation. Additionally, the ascites was directly observed by standard external Doppler ultrasound, as shown in Figure 5d and Video S5. The average volume of urine per hour increased by approximately 60 mL with pump actuation, which could be seen as the volume of ascites (Figure 5e). The experiment was conducted with continuous fluid infusion to maintain hemodynamic balance and avoid hydropenia.

To verify sensor operation after implantation, the flow rate was measured by a flexible wireless sensor and a commercial sensor individually. Since the commercial sensor detects the flow rate in a wired manner through the fluid traversal, the experiment was performed with the abdomen open. The results obtained by both sensors showed great consistency with the same narrow fluctuation range (Figure 5f). Although the variance of the flexible sensor was relatively high compared to that of the commercial sensor, its precision was sufficient to distinguish abnormalities from intrinsic flow. Thus, the flexible wireless sensor is a promising candidate for flow rate monitoring in implants.

To further characterize the artificial system after implantation, a long‐term experiment was performed. No obvious abnormal behavior was observed, which proved that the experimental subject did not suffer from severe pain. Since infection and ascites leakage has been reported to occur during the first 2–4 weeks for Alfapump,^41^ the soft implant shows superiority. The sensor function remained stable during the experiment period (Figures S8 and S9). Blood and urine biochemical indexes were analyzed after 4 weeks of implantation to understand the effect on hepatorenal function. In blood biochemical tests, all indexes of liver function decreased, especially AST, which implied that the artificial lymphatic system could partly take the load off the liver and benefit recovery of liver function. However, the increase in creatinine (Cr) indicated deterioration in kidney function, as reported by previous studies,^42^, ^43^ which can fully recover.^41^ In urine biochemical tests, the specific gravity and pH value changed slightly within the normal range, so no acute kidney impairment occurred during the experiment. The urinary calcium level declined by more than 50%, to slightly below normal. Since the urinary calcium level often fluctuates widely with the diet constitution, it is only a weak proof of kidney impairment. However, an increase in Cr was also found in urine biochemical results, which supported the hypothesis of kidney impairment.

To evaluate the long‐term biocompatibility, the tissue directly in contact with the artificial lymphatic system was sampled to obtain pathological sections, including the abdominal wall, urinary bladder and pouch for the pump cavity. The histopathological section of the urinary bladder is shown in Figure 5i, and Figure 5j presents an enlargement of the red rectangle in Figure 5i. The irregular hole in the middle of the image was where the outlet of the duct was inserted. The duct was covered by mass of connective tissue with inflammatory cell infiltration. Increased cellularity was observed at the implant site, which were most likely inflammatory cells. Similar conditions were observed in histopathological sections of the abdominal wall and pouch for the pump cavity (Figure S10). The results presented in histopathological sections showed mild aseptic inflammation, which usually occurred typically in the early stage of wound healing. Thus, the artificial lymphatic system is predicted to have great biocompatibility and stability.

## 3 CONCLUSION

We proposed a smart artificial lymphatic system that can transfer ascites as designed and monitor the real‐time flow rate wirelessly. The system can be actuated by an auxiliary external magnetic field to enlarge the controllable range of the flow rate. In addition to geometry optimization of the pump cavity, we established a theoretical model to analyze the efficient flow rate with respect to the actuation frequency. Additionally, the placement of the sensor was optimized, and the effectiveness of the system was verified by both in vitro experiments and in vivo experiments. Compared to previously reported treatment means, this system allows for long‐term low‐flow ascites transfer and flow rate monitoring, which can compensate for pathogenically insufficient or impaired lymphatic function. With the flow rate perception, the system can detect and warn of potential abnormalities. In addition, the implant of this system is totally free of battery and gear, thus bringing high reliability from the start. The liver function of the experiment animal was improved and severe complications did not occur for 4 weeks based on the histopathological section. The recovery of experiment animal confirmed the function of the artificial lymphatic system. The palliation of the real patient during the implantation period is also promising. If the artificial lymphatic system is used for ascites treatment or as palliation of patients with advanced liver cancer, it can be implanted for an extended period. Future work includes the integration of an external actuation source and a detection system as a wearable device, which can supplant the costly VNA apparatus and liberate patients from immobile treatment procedures. Furthermore, the actuation and detection method could be analyzed to find a more efficient method with high accuracy. Additionally, future work will focus on adaptation of the artificial system to other applications. For example, the system could be used as a solution to encephalic and pleural effusion or as an artificial vessel to improve the local blood supply.

## 4 MATERIALS AND METHODS

### 4.1 Sensor fabrication

#### 4.1.1 Fabrication of silicon micromolds

A thin layer of Cu was first deposited on a precleaned silicon (100) wafer. The Cu layer was etched into periodic square patterns in the diluted etching solution with a photoresist mask spun over it. Afterward, the mask was sequentially cleaned by acetone, ethanol and deionized water. The silicon wafer was further etched in a KOH solution (250 g of KOH, 250 g of IPA, 800 g of H_2_O) at 80°C with the patterned Cu mask for protection. Then, the wafer was moved into the diluted etching solution again to remove the protective Cu mask and further cleaned by deionized water. Finally, the silicon mold was treated with 1H,1H,2H,2H‐perfluorooctyltriethoxysilane in a vacuum chamber for 15 h.

#### 4.1.2 Sensor assembly

Layers of Ni (10 nm thick) and Au (150 nm thick) were successively deposited on a silicon wafer with layers of PMMA and PI spun on the surface. With a patterned photoresist mask, the metal layers were etched as designed to form a capacitance and coils. Excessive PI was removed by reactive ion etching, and the coils were assembled in an asymmetrical mode with a PDMS insulation layer in the middle. Then, the laminate was transfer printed by a stamp onto a flexible PDMS substrate. The micropyramid layer was fabricated by liquid PDMS solidified over the micromolds with a release agent and placed on the electrodes to form changeable capacitances. The structure assembled as mentioned above was eventually encapsulated by PDMS films. The elongation of stretchability breaking point was measured by 8%, meeting the requirement of stretchability^44^ and the twisting limit was more than 180° (Figures S11 and S12).

### 4.2 Artificial lymphatic system assembly

A half‐ellipsoid mold defined the hollow geometry of the pump cavity, and each end of the mold was supported by a concentric mold to define the exterior geometry of the pump cavity. The molds were made of 400C stainless steel, the same as that of a scalpel, to avoid the introduction of extra allergens. Then, biocompatible silica gel was injected into and cured in the assembled molds to fabricate elements of the pump cavity. Afterward, two elements were bound by silica gel to assemble a pump cavity. The duckbill valves were also made of biocompatible silica gel by injection molding and connected to the pump cavity by medical‐grade ducts. A magnetic tablet was attached to the surface of the pump cavity with a biocompatible binder and encapsulated by silica gel. The magnetic tablet attached to the pump cavity is made of NbFeB with the magnetic strength of 0.3 T and coercivity of 796 kA/m. Flexible sensors were wrapped around the outlet of valves.

### 4.3 In vitro tests

#### 4.3.1 Wireless measurement setup

Pressure was applied onto the pressure‐sensitive region by a mechanical testing machine (Zwick IBTC‐300SL). The capacitance of the sensor was measured with a Keysight Precision LCR meter. The sensor was characterized at its series resonance frequency to avoid other interference issues. Wireless measurements were conducted with a VNA (E5071C Keysight, Agilent) and a reader coil (same as the coil of the sensor) to measure *S*_11_. The inductance of the reader coil was 6.93 pH. Since the capacitive sensors have been proved to be temperature‐independent, measurements were conducted with the temperature (25 ± 1°C) and atmospheric humidity (50 ± 10% relative humidity) controlled. The actuation was supplied by a standard gear pump for calibration. The resonance frequency shifts were recorded by a high‐speed camera.

#### 4.3.2 In vitro system function assessment

The pump cavity was fixed onto the platform and actuated by an external device above it. The external device is a combination of an electromagnet and a fan to disperse the heat (Figure S13). The actuation was set as a square wave with an amplitude of 24 V. Deionized water was chosen as the ascites simulant, and the inlet and outlet of the artificial system were both placed in open containers. The magnetic strength of the external electromagnet is measured at 0.8 T. The distance between the coil and pump was set in the range of 10–20 mm to demonstrate the flow velocity response to the coupling distance, and it was set as 10 mm when the flow velocity response to lift was analyzed.

### 4.4 In vivo tests

#### 4.4.1 In vivo system function assessment

In vivo animal experiments were performed at Medical Services Biotechnology Co., Ltd. (Beijing, China) and approved by the Ethics Committee of Medical Services Biotechnology Co., Ltd. (MDSW‐2021‐080A).

Bama miniature pigs (female, 31.7 kg ± 9.6 kg) were used in compliance with the regulations of the animal care and use committee of our hospital. One animal was used for in vivo function verification, and three animals were used for long‐term verification. Only female experiment animals were chosen since no evidence proved that the treatment of ascites is gender‐related based on previous animal experiments and clinical research.^9^, ^45^, ^46^ The implantation surgery was conducted under isoflurane inhalation anesthesia by laparotomy. The pump cavity was placed inside a pouch beneath the skin and fixed with sutures, and the wireless sensor was implanted by a similar procedure. The distance between the external magnet and pump was measured at 11.2 mm. The outlet duct was inserted into the urinary bladder through a cut and sutured for fixation with the connection clapped. Then, normal saline was injected from the inlet to remove the air, and the connection was clapped when the system was filled with fluid. The inlet was inserted and fixed in the lower abdomen to ensure the submersion into the ascites. Finally, the two parts were connected after removal of air. The external device was placed close to the pump cavity, and the location was found by a Hall element. Doppler ultrasound (Shenzhen Mindray Bio‐Medical Electronics Co., Ltd., DC‐75) was used to check the flow and clearance of the ducts. For wireless sensor testing, the reader coil was placed on the skin directly above the internal sensor. For commercial sensor contrast, the commercial sensor was linked to the system and performed detection in a wired manner.

#### 4.4.2 Blood and urine biochemistry test

For blood biochemistry tests, blood was collected before and after long‐term in vivo experiments. After the blood samples were obtained, the supernate was extracted after 10 min of centrifugation at a speed of 3000r inside a benchtop high‐speed refrigerated centrifuge (Beijing DLAB Scientific Co., Ltd., D3024R). Then, the supernate was stored at 4°C until testing. Different indexes were measured by corresponding determination kits. For urine biochemistry tests, the urine samples were analyzed by an analyzer (URIT Medical Electronic Co., Ltd., Guilin, URIT‐500B) after collection, and the indexes were obtained.

#### 4.4.3 Histopathological examination

Fresh tissue samples were fixed with 4% paraformaldehyde solution for more than 24 h and checked before examination. The samples were removed from the fixation solution, and the target tissue was trimmed with a scalpel in a ventilation cupboard. The trimmed tissue was dehydrated with gradient ethanol (Sinopharm Group Chemical Reagent Co., Ltd.) inside a dehydration box and finally immersed in melted paraffin for 1 h three times at 65°C. The paraffin‐soaked sample was placed into an embedding frame (Wuhan Junjie Electronics Co., Ltd., JB‐P5) filled with melted paraffin and cooled over a frozen platform (Wuhan Junjie Electronics Co., Ltd., JB‐L5) at −20°C. The cooled paraffin bulk was removed and trimmed. The trimmed paraffin bulk was sliced into 4 μm‐thick sections and flattened on the surface of water at 40°C inside a tissue spreader (Zhejiang Kehua Instrument Co., Ltd., KD‐P). The slices were picked up with glass slides and heated at 60°C to remove residual moisture. Then, the slices could be kept at room temperature. The slices were successively rinsed with xylene, ethanol and deionized water to remove protective paraffin. Then, the slices were successively dyed with hematoxylin solution, hematoxylin differentiation solution and hematoxylin Scott Tap Bluing, with rinsing after each step. Afterward, the slices were dehydrated and dyed with eosin for 5 min. Eventually, the slices were dehydrated again with gradient ethanol and sealed with neutral gum. The target tissue slices were observed with a microscope, and images were acquired and analyzed.

## AUTHOR CONTRIBUTIONS

**Peng Wang:** Investigation (lead); methodology (equal); visualization (lead); writing – original draft (lead). **Ji Fu:** Funding acquisition (equal); writing – review and editing (equal). **Peng Jin:** Investigation (supporting); methodology (supporting). **Jin Zeng:** Investigation (supporting). **Xiaohui Miao:** Visualization (supporting). **Heling Wang:** Methodology (supporting); writing – review and editing (supporting). **Yinji Ma:** Funding acquisition (equal); methodology (supporting); writing – review and editing (supporting). **Xue Feng:** Funding acquisition (equal); resources (lead); supervision (lead); writing – review and editing (equal).

## CONFLICT OF INTEREST STATEMENT

The authors declare no conflicts of interest.

### PEER REVIEW

The peer review history for this article is available at https://www.webofscience.com/api/gateway/wos/peer-review/10.1002/btm2.10567.

## Supporting information


**Data S1:** Supporting Information.

**Video S1:** Supporting Information.

**Video S2:** Supporting Information.

**Video S3:** Supporting Information.

**Video S4:** Supporting Information.

**Video S5:** Supporting Information.

## Data Availability

The data that support the findings of this study are available in the supplementary material of this article.
