# Supplementary material for: A soft, bioinspired artificial lymphatic system for interactive ascites transfer
Source: Bioeng Transl Med. 2023 Aug 3;8(5):e10567. doi: 10.1002/btm2.10567 (PMC10486333; doi:10.1002/btm2.10567)
Supplement: Supplementary file 1 — Data S1: Supporting Information. [file BTM2-8-e10567-s002.docx]

**Supplementary Note 1** Equivalent circuit model

The motion of the magnetic tablet can be seen as a mass motivated by square waves coupled with a spring abstracted from the membrane elasticity. The equation of motion can be described as Eq. 1, where m and k refer to the equivalent mass and stiffness, respectively. f(t) is the square wave with a frequency of T. K and U refer to the coupling parameter and amplitude of the input voltage.

$\left. m\ddot{x}+kx=KUf(t \right)$ (1)

Since the square wave can be decomposed by Fourier transform, the motion of the magnetic tablet can be seen as the sum of the responses to a sequence of simple harmonic exciting forces. Thus, the displacement of the magnetic tablet is expressed as Eq. 2, where $\omega_{n}$ and $\omega_{i}$ are the intrinsic frequency and the frequencies of the harmonic exciting forces.

$x=\frac{4}{\pi}\sum_{i=1}^{\infty} \frac{1}{i}\frac{KU}{\left. {m(\omega}_{n}^{2}-\omega_{i}^{2} \right)}\left( 1-\frac{\omega_{i}}{\omega_{n}} \right)\sin\left( \omega_{i}t \right)$ (2)

The pump is actuated by displacement of the magnetic tablet and can thus be viewed as a current source in the fluidic circuit model. The input current $Q_{in}\left( t \right)$ can be expressed as Eq. 3.

$Q_{in}\left( t \right)=S\frac{dx}{dt}$ (3)

According to the mass conservation law, the relationship between the input current and the abdominal pressure $P_{a}$ can be derived as

$Q_{i}\left( t \right)=\frac{{P_{a}-P}_{out}}{R_{1}}+Q_{in}$ (4)

In Eq. 4, $R_{1}$ is the fluidic resistance of the inlet duct, and $Q_{i}\left( t \right)$ is the outlet flow rate.

In addition, the output pressure $P_{out}$ can be expressed as a function of the outlet flow rate through the membrane compliance of the urinary bladder ($C_{f2}$), as shown in Eq. 5.

$P_{out}=R_{2}Q_{i}+\frac{1}{C_{f2}}\int Q_{i}\left( t \right)dt$ (5)

Similarly, the abdominal pressure $P_{a}$ is affected by the membrane compliance of the abdomen cavity ($C_{f1}$) and expressed as follows:

$P_{a}=P_{a0}-\frac{1}{C_{f0}}\int Q_{i}\left( t \right)dt$ (6)

In Eq. 6, $P_{a0}$ is the initial condition of the abdominal pressure.

Using Eqs. 2-6, the following equation can be derived by converting the electric actuation $\left. Uf(t \right)$ into the flow rate $Q_{i}\left( t \right)$:

$\left( 1+\frac{R_{2}}{R_{1}} \right)\frac{{dQ}_{i}}{dt}+\frac{1}{R_{1}}\left( \frac{1}{C_{f0}}+\frac{1}{C_{f2}} \right)Q_{i}=-\frac{4}{\pi}\frac{KUS}{m}\sum_{i=1}^{\infty} \frac{1}{i}\frac{\omega_{i}}{\omega_{n}\left( \omega_{n}+\omega_{i} \right)}\sin\left( \omega_{i}t \right)$ (7)

Thus, the flow rate of the system is analytically derived as follows:

$Q_{i}=-\frac{4}{\pi}\frac{KUS}{m}R_{1}\sum_{i=1}^{\infty} \frac{\omega_{i}}{\left. {\omega_{n}(\omega}_{n}^{2}-\omega_{i}^{2} \right)}\frac{1}{\sqrt{1+\omega_{i}^{2}\tau^{2}}}\sin(\omega_{i}t-\alpha)$ (8)

In Eq. 8, $\tau$ indicates the time constant, which is expressed as $\tau=\frac{C_{f0}+C_{f2}}{C_{f0}C_{f2}}(R_{1}+R_{2})$, and $\alpha=\tan^{-1} (\omega_{i}\tau)$.

Considering the effect of the duckbill valves, the flow rate should be nonnegative. Therefore, the flow rate of the system is expressed as Eq. 9.

$Q_{i}=\left\{ \begin{aligned} -\frac{4}{\pi}\frac{KUSR_{1}}{m}\sum_{i=1}^{\infty} \frac{\omega_{i}}{\left. {\omega_{n}(\omega}_{n}^{2}-\omega_{i}^{2} \right)}\frac{1}{\sqrt{1+\omega_{i}^{2}\tau^{2}}}\sin(\omega_{i}t-\alpha) \frac{\alpha}{\omega_{i}}-\frac{T}{2}<t<\frac{\alpha}{\omega_{i}} \\ 0 \frac{\alpha}{\omega_{i}}<t<\frac{\alpha}{\omega_{i}}+\frac{T}{2} \end{aligned} \right.$ (9)


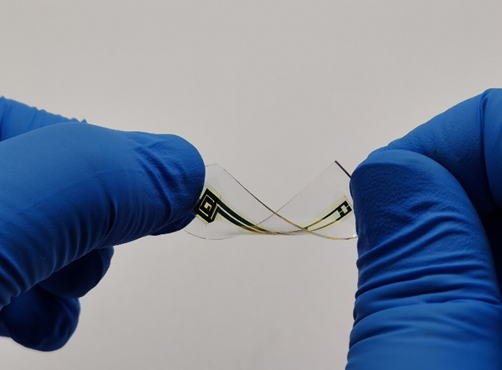


**Supplementary Fig. 1** Optical image of the flexible sensor when bent and twisted, showing great mechanical robustness and flexibility.


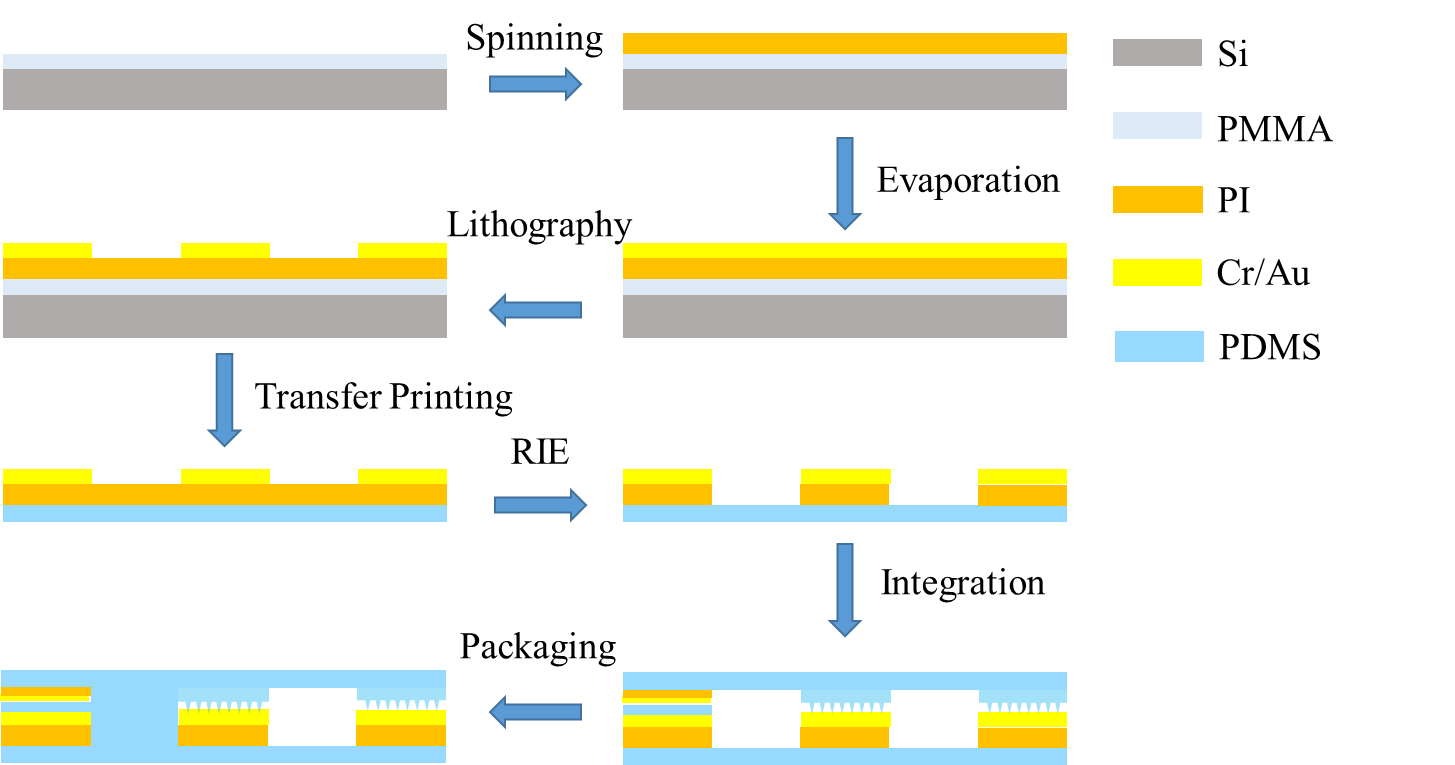


**Supplementary Fig. 2** Fabrication of the flexible sensor.


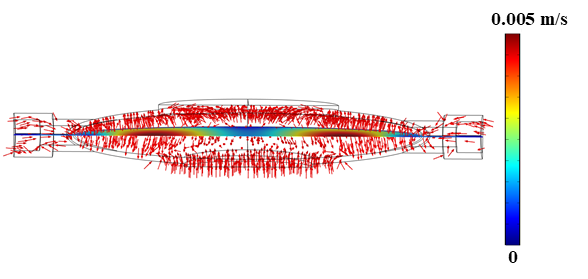


**Supplementary Fig. 3** 3D fluid velocity field calculated by the FEM (COMSOL 5.6).


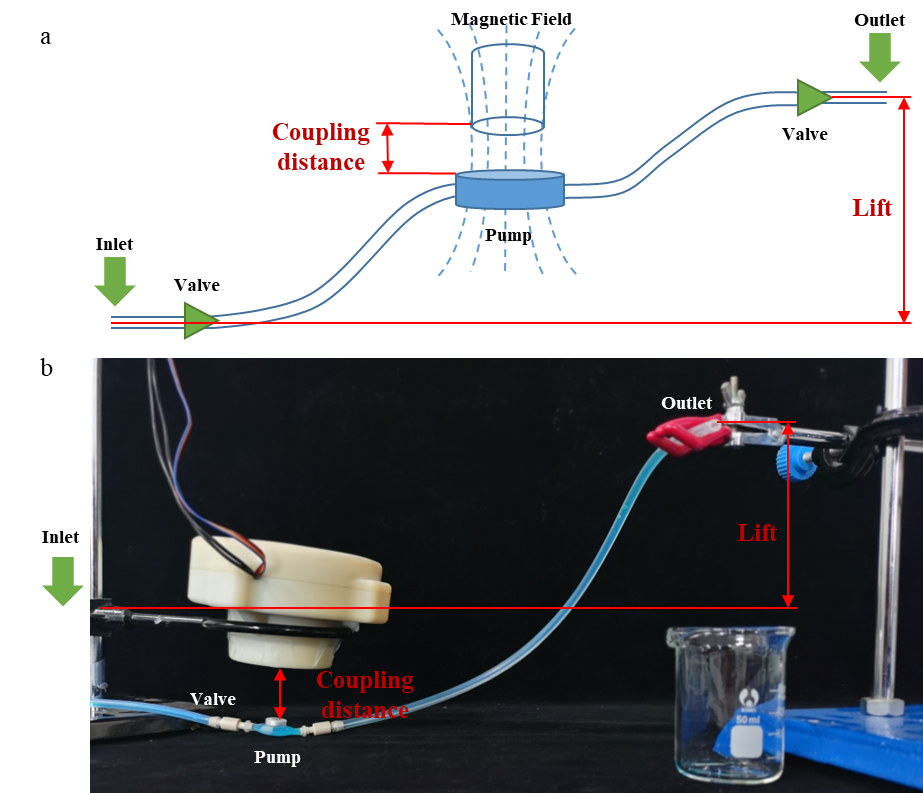


**Supplementary Fig. 4** Illustration of the lift and coupling distance for the artificial lymphatic system.

Fig. r1 Temperature distribution before and after the sensor worked.
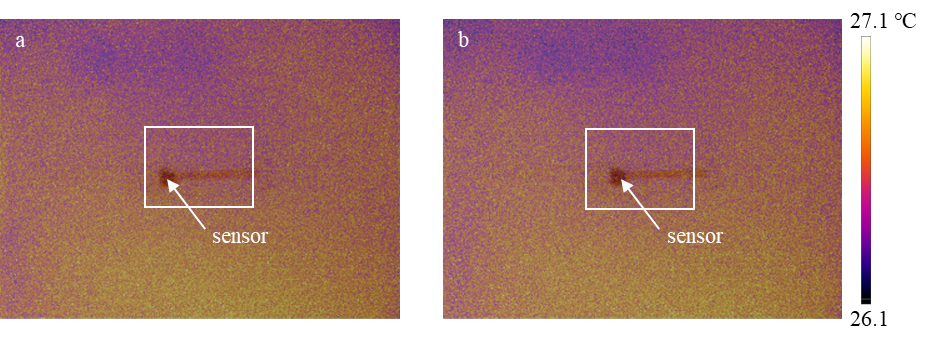


**Supplementary Fig. 5** Temperature distribution before and after the sensor worked.


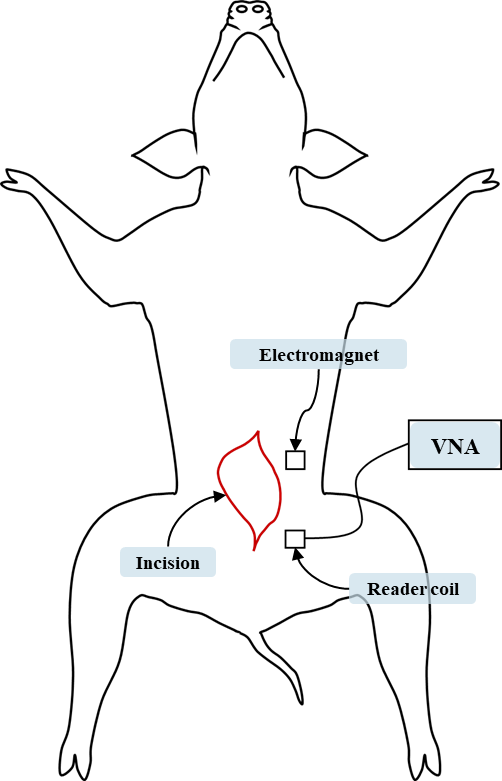


**Supplementary Fig. 6** Illustration of the experimental setup in a pig. The incision in this image refers to Fig. 5a. The electromagnet was placed externally close to the pump cavity. The reader coil was connected to a VNA for data collection.


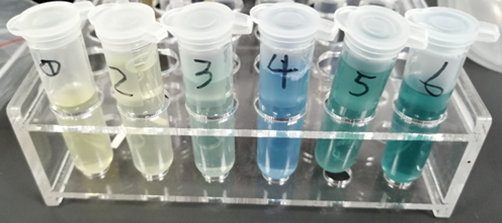


**Supplementary Fig. 7** Indigo carmine appearance in urine samples. 1, 2: urine sample before transfer; 4: blank contrast of indigo carmine dissolved in peritoneal dialysis solution; 3, 5, and 6: urine sample after transfer for 1 h, 2 h, and 3 h, respectively.



**Supplementary Fig. 8** Continuous monitoring result





**Supplementary Fig. 9** Drift over four weeks


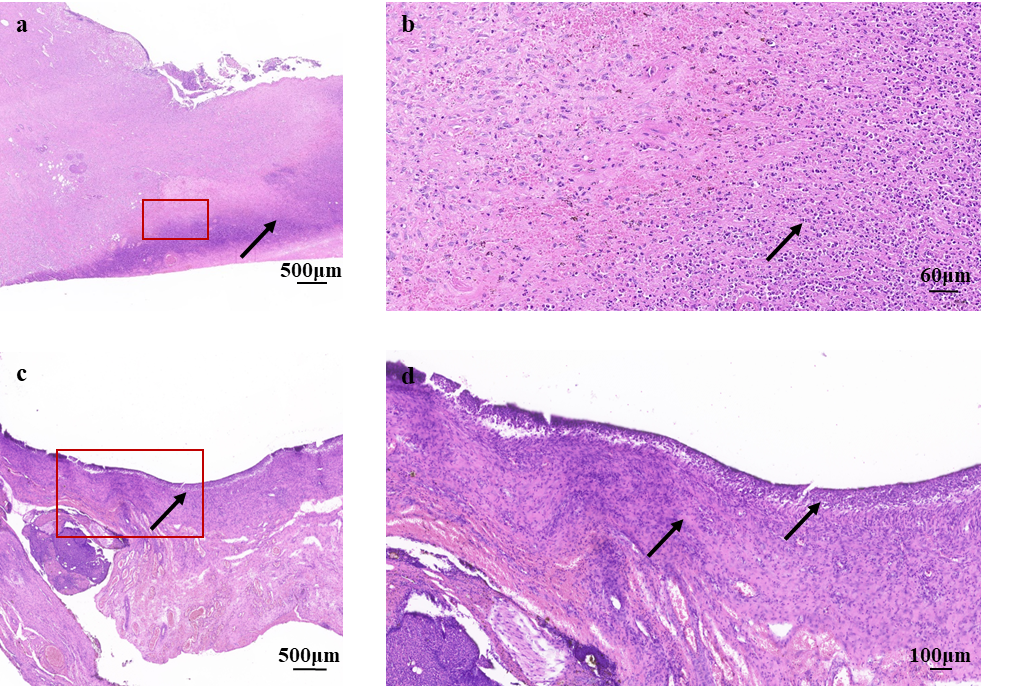


**Supplementary Fig. 10 a**,**b**, Histopathological section of pouch for the pump cavity. **b** presents an enlargement of the red rectangle in **a**. **c**,**d**, Histopathological section of the abdominal wall. **d** presents an enlargement of the red rectangle in **c**. The pump cavity was covered by a great deal of connective tissue with inflammatory cell infiltration (black arrows).


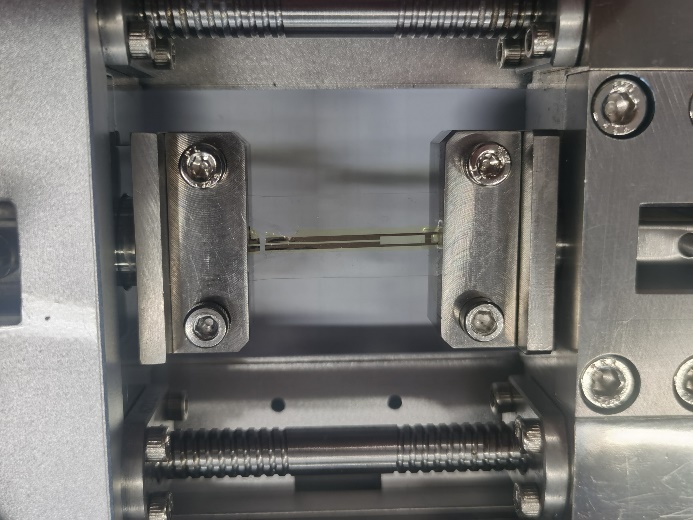


**Supplementary Fig. 11** The sensor after stretchability breaking point test


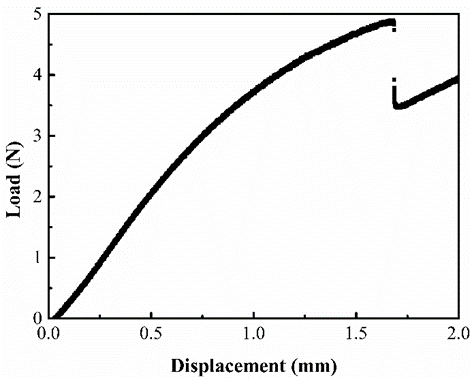


**Supplementary Fig. 12** Relationship between load and displacement


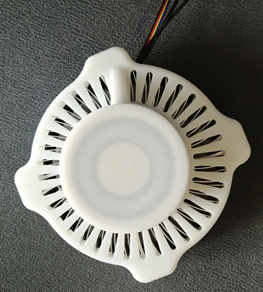


**Supplementary Fig. 13** Photo of external device
